# Colonisation of Newborn Piglets with a Mixture of *Bacteroides* Species Improves Their Gut Health and Performance

**DOI:** 10.3390/microorganisms13102356

**Published:** 2025-10-14

**Authors:** Jitka Matiasovicova, Katerina Nechvatalova, Daniela Karasova, Alena Sebkova, Jan Matiasovic, Jiri Volf, Vladimir Babak, Ivan Rychlik

**Affiliations:** Veterinary Research Institute, 621 00 Brno, Czech Republic; matiasovicova@vri.cz (J.M.); nechvatalova@vri.cz (K.N.); karasova@vri.cz (D.K.); asebkova@vri.cz (A.S.); matiasovic@vri.cz (J.M.); volf@vri.cz (J.V.); babak@vri.cz (V.B.)

**Keywords:** sow, piglet, gut microbiota, *Bacteroides*, *Prevotella*, *Clostridium perfringens*, probiotic

## Abstract

Due to the low populations of *Bacteroides* sp. in the gut microbiota of sows compared to nursed piglets, sows may not represent an ideal source of *Bacteroides* sp. for newborn piglets. In this study, we therefore tested the effect of oral administration of a mixture of *Bacteroides thetaiotaomicron*, *Bacteroides vulgatus*, *Bacteroides fragilis* and *Bacteroides xylanisolvens* on the microbiota development of newborn piglets. Oral administration of such a mixture to piglets within 12 h after parturition did not result in any adverse effects. Sequencing of 16S rRNA showed that 4 days after administration, these species formed approx. 20% of total faecal microbiota and affected the development of gut microbiota in treated piglets. The treatment resulted in an increased abundance of *Veillonella caviae*, *Fusobacterium gastrosuis*, *Dialister* sp., *Clostridium jeddahitimonense*, *C. cadaveris*, *Butyricicoccus pullicaecorum*, *Actinobacillus indolicus*, *A. minor*, *Streptococcus pasteurianus*, *S. parasuis*, *S. equinus*, *S. pluranimalium*, *S. thoraltensis* and *S. suis*. On the other hand, administration of the *Bacteroides* mixture suppressed piglet colonisation by *C. disporicum* and multiple species from family Prevotellaceae. *Bacteroides*-treated piglets exhibited significantly higher body weight than untreated controls at 3 months of age. Administration of a mixture of *Bacteroides* shaped the development of gut microbiota in nursed piglets, which resulted in improved parameters at the end of the pre-fattening period.

## 1. Introduction

Mammals are born nearly sterile, and microbial colonisation of their bodies begins immediately after birth. The densest microbial populations can be found in the intestinal tract, which changes from nearly zero bacterial counts at birth to 10^10^ CFU/g only a few days later. Previous studies on the gut microbiota of omnivorous, warm-blooded animals reported that around one thousand different bacterial species colonise the intestinal tract [1,2,3], the vast majority of which are commensal or even beneficial for its host. Despite this, if live bacterial cultures are tested as probiotics, findings on the diversity of gut microbiota are ignored and only a small subset of gut microbiota members such as Lactobacilli, Bifidobacteria, Enterococci, or Bacilli are tested repeatedly [4,5,6,7,8]. However, there is no reason why the remaining gut microbiota members should not be tested for their probiotic potential as well, and the first reports of this type have begun to appear in pigs [9] and chickens [10].

In our earlier studies, we characterised the microbiota of sows and nursed and weaned piglets and found that the first colonisers of newborn piglets included *E. coli* and *Fusobacterium* [11,12]. *Clostridium perfringens* was also a common coloniser of newborn, one-day-old piglets. Within a few days during the first week of life, representatives of genus *Bacteroides* appear [11] and remain present until weaning, when these are replaced with taxonomically related *Prevotella* species [13,14,15]. This indicates that breast milk feeding supports *Bacteroides* colonisation while conventional feed formula supports the proliferation of *Prevotella* species. As a consequence, *Bacteroides* are present at a low abundance in adult pigs, including sows, which therefore do not represent an ideal source of *Bacteroides* for newborn piglets. It can be argued that sows colonised with *Bacteroides* at a low abundance may still act as a source of *Bacteroides*, especially if these are then positively selected by a milk diet. However, if *Bacteroides* were administered to newborn piglets experimentally immediately after birth, *Bacteroides* colonisation could be accelerated, all piglets in the litter would be equally colonised from the first hours of their life and the whole litter would develop uniformly. In addition, when we retrospectively checked previously published data, we noticed that when *Clostridium perfringens* formed around 10% of total microbiota of one-day-old piglets, *Bacteroides* species were present at a low abundance. However, when *Bacteroides* abundance increased to 20% of total microbiota in four-day-old piglets, *C. perfringens* decreased to approx. 1% of total microbiota [11]. This observation led to the current study, in which we tested a mixture of *Bacteroides* species as probiotics for newborn piglets. Besides specific interest in *Bacteroides* species as probiotics, this study has been designed also as a proof of concept, assessing whether other bacterial species from the intestinal tract can be considered as novel types of probiotics in addition to the traditional lactic acid bacteria.

## 2. Materials and Methods

### 2.1. Ethics Statement

The handling of animals in this study was performed in accordance with current Czech legislation (Animal Protection and Welfare Act No. 246/1992 Coll. of the Government of the Czech Republic). The specific experiments were approved by the Ethics Committee of the Veterinary Research Institute, followed by the Committee for Animal Welfare of the Ministry of Agriculture of the Czech Republic on 3 March 2023 (permit number MZe 2406).

### 2.2. Bacterial Strains and Culture

To obtain bacterial strains for this study, rectal swabs were collected from piglets 1–7 days of age at farm A, where the majority of subsequent experiments were performed, and transported on ice to the laboratory for further processing within one hour. The swabs were resuspended in 5 mL of PRAS (0.1 g magnesium sulphate heptahydrate, 0.2 g monobasic potassium phosphate, 0.2 g potassium chloride, 1.15 g dibasic sodium phosphate, 3.0 g sodium chloride, 1.0 g sodium thioglycolate, 0.5 g L-cysteine and 1000 mL distilled water; final pH 7.5 ±  0.2 at 25 °C) in an anaerobic chamber. In the next step, the samples were serially diluted and plated on Wilkins-Chalgren anaerobe agar (Oxoid, Basingstoke, UK) supplemented with 5 mg/L hemin, 1 mg/L cellobiose, 0.5 g/L soluble starch, 1 mg/mL maltose, 0.2 mL vitamin K1 solution (0.1 mL of filter sterilized vitamin K1 in 20 mL 95% ethanol) and 0.5 mg/mL L-cysteine. The plates were incubated in an anaerobic chamber under an atmosphere consisting of 10% CO_2_, 5% H_2_ and 85% N_2_ at 37 °C for 48 h. Approx. 10 well-separated colonies of different morphology were selected from each agar plate, subcultured and tested for growth under aerobic conditions. Aerobically growing colonies were discarded and isolates of strict anaerobes were stored at −70 °C in BHI medium (Oxoid, Basingstoke, UK) containing glycerol at 10% concentration. To assign obtained isolates to bacterial species, DNA purified from strict anaerobes using a DNeasy Blood & Tissue Kit (Qiagen, Hilden, Germany) was used as a template in the PCR amplifying over the whole 16S rRNA gene using forward TGAAGAGTTTGATCATGGCTCAG and reverse AGGAGGTGATCCAGCCGCA primers. The resulting PCR products were subjected to Sanger sequencing with the forward primer. Obtained sequences were BLAST compared with the GenBank database (https://blast.ncbi.nlm.nih.gov/Blast.cgi, accessed on 10 October 2025) and isolates belonging to genus *Bacteroides*, *Lactobacillus* and *Bifidobacterium* were identified. DNA from such isolates was then subjected to whole genome sequencing using an external service.

The whole genomic sequences were annotated with RAST [16] and genomes of individual *Bacteroides* species were searched for the most frequent antibiotic resistances, i.e., *tetQ* and *linA* [17,18]. Presence of *tetQ* in the genome of *Bacteroides thetaiotaomicron* An878 was the reason for its replacement with *Bacteroides thetaiotaomicron* ET42 (see below). Later, the search for horizontally acquired antibiotic resistance genes was expanded using ABRicate and combined results of the ARGANNOT, CARD, MEGARES, NCBI and RESFINDER databases [19,20,21,22]. After such analysis, isolates of *B. thetaiotaomicron*, *Bacteroides vulgatus*, *Bacteroides fragilis*, *Bacteroides xylanisolvens*, *Bifidobacterium boum*, *Lactobacillus ruminis*, *Lactobacillus salivarius* and *Lactobacillus mucosae* were selected for subsequent experiments.

### 2.3. Animal Experiments

The first three tests were performed at the same farm from which all *Bacteroides* strains were cultured and the tests focused on the ability of the used strains to colonise and their safety. This farm routinely used intramuscular amoxycillin administration to control neonatal diarrhoea. The fourth experiment was performed at another farm which did not use antibiotics when testing the *Bacteroides* probiotic product.

The first test was performed with 15 piglets, five of which were orally inoculated with a mixture of *Bacteroides*, *Lactobacillus* and *Bifidobacterium*, five piglets which were given a mixture of *Bacteroides*, *Lactobacillus* and *Bifidobacterium* together with amoxycillin treatment and the remaining five piglets were treated with amoxycillin only. When performing the first experiment, *Bacteroides fragilis* used in later experiments was not yet available so the piglets were treated with a mixture of 3 *Bacteroides* species only, i.e., *B. thetaiotaomicron* An878, *B. vulgatus* An905 and *B. xylanisolvens* An931 and *Bifido. boum* An918, *L. ruminis* An917, *L. salivarius* An879 and *L. mucosae* An939 (Table 1).

In the second experiment, 15 piglets were treated with amoxycillin, 5 piglets were treated with both *Bacteroides* mixture and amoxycillin and 9 piglets were treated with the *Bacteroides* mixture only. The original *B. thetaiotaomicron* An878 was replaced with another strain of the same species, *B. thetaiotaomicron* ET42, because of the absence of the *tetQ* gene in the genome of the ET42 strain (Table 2). In addition, since *B. fragilis* ET48 was obtained in the meantime, these piglets were treated with a mixture of 4 *Bacteroides* species, i.e., *B. thetaiotaomicron* ET42, *B. vulgatus* An905, *B. xylanisolvens* An931 and *B. fragilis* ET48. Since none of the tested Lactobacilli and *Bifido. boum* colonised piglets in the first experiment (see below), these strains were skipped from the remaining tests. The same mixture of 4 different *Bacteroides* species was then used in all remaining experiments.

In experiment 3, the microbiota of 12 piglets treated with amoxycillin, 3 piglets treated with both a *Bacteroides* mixture and amoxycillin and 12 piglets treated with a *Bacteroides* mixture only was characterised.

Experiment 4 was performed on another farm in which 40 piglets were treated with a *Bacteroides* mixture and 50 piglets were used as controls without any treatment. Piglets were born in weekly intervals at this farm and 10 piglets born within one week were treated with a mixture of *Bacteroides*. One week later, all piglets remained untreated and 10 piglets were again treated in the following week, etc. Since the whole test was started and finished with non-treated piglets, we compared 5 production weeks of control piglets without any treatment with 4 production weeks of piglets treated with a *Bacteroides* mixture. Out of all piglets, only 11 *Bacteroides*-treated and 10 control piglets were sampled for microbiota composition, while the rest of the piglets were used for comparison of production parameters at the end of the pre-fattening period when the piglets were 3 months of age.

To prepare probiotic mixtures for newborn piglets, all *Bacteroides*, *Lactobacillus* and *Bifidobacterium* species were grown individually in BHI Vegan medium (HiMedia, Thane, Maharashtra, India) in an anaerobic cabinet at 37 °C for 48 h. When the culture was completed, bacterial counts were approx. 1 × 10^8^ CFU/mL. Equal volumes of each of the 4 cultures (7 cultures in the first experiment) were mixed and immediately delivered to the farm in multiple aliquots. The mixtures were kept at 4 °C for a maximum of 5 days and any opened aliquot was used completely. Approx. 10 h old piglets were treated individually by oral administration of 1 mL of the mixture of all strains. Efficiency of colonisation was tested by collecting rectal swabs of 4-day-old piglets.

### 2.4. 16S rRNA Sequencing

The samples were homogenized in a MagNALyzer (Roche, Basel, Switzerland). Following homogenization, the DNA was extracted using a QIAamp DNA Stool Mini Kit according to the manufacturer’s instructions (Qiagen, Hilden, Germany) and the DNA concentration was determined spectrophotometrically. DNA samples were diluted to 5 ng/mL and were used as a template in polymerase chain reaction (PCR) with forward primer 5′-TCG TCG GCA GCG TCA GAT GTG TAT AAG AGA CAG-MID-GTC CTA CGG GNG GCW GCA G-3′ and reverse primer 5′-GTC TCG TGG GCT CGG AGA TGT GTA TAA GAG ACA G-MID-GTG ACT ACH VGG GTA TCT AAT CC-3′. MIDs shown above represent different sequences 5, 6, 7 or 9 base pairs in length that were used to identify individual samples within the sequencing groups. PCR amplification was performed using a HotStarTaq Plus Master Mix kit (Qiagen, Hilden, Germany) and the resulting PCR products were purified using AMPure beads (Beckman Coulter, Brea, CA, USA). In the next step, the concentration of PCR products was determined spectrophotometrically, the DNA was diluted to 100 ng/µL and groups of 14 PCR products with different MID sequences were indexed with the same indices using a Nextera XT Index Kit (Illumina, San Diego, CA, USA). Prior to sequencing, the concentration of differently indexed samples was determined using a KAPA Library Quantification Complete kit (Kapa Biosystems, Wilmington, MA, USA). All indexed samples were diluted to 4 ng/µL and 20 pM phiX DNA was added to a final concentration of 5% (*v*/*v*). Sequencing was performed using a MiSeq Reagent Kit v3 and MiSeq apparatus (Illumina, San Diego, CA, USA).

Analysis of sequencing data was performed with QIIME 2 [23]. Raw sequence data were demultiplexed, quality filtered and sequencing primers were clipped using Je [24] and fastp [25]. The resulting sequences were denoised with DADA2 [26]. Taxonomy was assigned to ASVs using the q2-feature-classifier [27] and classify-sklearn naïve Bayes taxonomy classifier against the Silva 138 [28]. All the software tools were used with default settings.

### 2.5. Statistics

To identify key events following *Bacteroides* mixture administration, 5 different groups of piglets were defined for downstream analyses. The first group was formed by all amoxycillin treated piglets at farm A, hereafter designated as ATB (antibiotic treated). The second group comprised all piglets from farm A treated both with *Bacteroides* mixture and amoxycillin, hereafter called PA (probiotics and antibiotics). The third group comprised piglets from farm A treated with *Bacteroides* mixture only, hereafter called P1 (probiotics 1). Two groups of piglets from farm B were designated as CTRL (control, i.e., no antibiotics or probiotics) and P2 for piglets provided probiotic *Bacteroides* mixture at farm B.

Identification of differently abundant taxa was performed by PERMANOVA with 10,000 permutations and using Euclidian matrix distances (R-project, package vegan, Adonis function followed by pairwise comparisons). Only ASVs which formed more than 0.1% of total microbiota in at least one piglet and which were present in at least 15 piglets (out of 93 subjected to 16S rRNA sequencing in the study) were subjected to PERMANOVA analysis. Following PERMANOVA, significant differences (*p* < 0.05) were picked up from comparisons between ATB and PA groups, ATB and P1 groups and CTRL and P2 groups. Differences in microbiota composition between PA and P1 groups were considered as well.

## 3. Results

### 3.1. Safety of the Tested Mixtures

Oral administration of tested *Bacteroides* mixtures to newborn piglets did not lead to any adverse responses and all strains tested in this study can be therefore considered as safe.

### 3.2. Gut Microbiota Composition Following Administration of a Bacteroides Mixture

**Figure 1:**
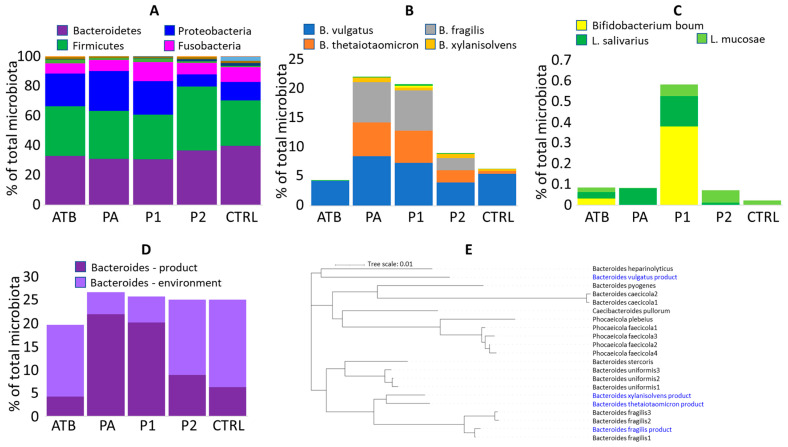
Colonisation of newborn piglets. (**A**) Microbiota composition of ATB, PA and P1 piglets from farm A and P2 and CTRL piglets from farm B at phylum level. (**B**) Abundance of used strains in rectal microbiota of 4-day-old piglets. (**C**) Zoom in *Lactobacillus* and *Bifidobacterium* colonisation. Mind the Y axis scaling in (**B**,**C**). Apparently, the increased abundance of *Bifido. boum* in P1 piglets was affected by a single highly positive piglet. (**D**) Summed abundance of *Bacteroides* species from the used mixture and those of environmental origin. (**E**) Relatedness of *Bacteroides* strains used in the mixture and those of environmental origin found in control piglets.

Microbiota composition in 32 piglets in the ATB group, 14 piglets in the PA group, 26 piglets in the P1 group, 10 piglets in the P2 group and 11 piglets in the CTRL group was characterised by 16S rRNA sequencing. The sample with the lowest coverage was characterised by 28,402 sequences, while 239,953 sequences were available for the sample with the highest coverage. Microbiota composition at the phylum level showed a distribution typical for the age group [11], i.e., dominating Bacteroidetes and Firmicutes, followed by Proteobacteria and Fusobacteria, and administration of *Bacteroides* product did not lead to extensive differences in the composition of gut microbiota at the phylum level (Figure 1A).

### 3.3. Ability of Used Strains to Colonise

Except for *B*. *vulgatus*, the remaining *Bacteroides* strains were detected in the treated piglets at a significantly higher abundance than in the non-treated controls (Figure 1B). Absence of significant colonisation of *B. vulgatus* was caused by a high abundance of *B. vulgatus* of environmental origin also in the microbiota of ATB and CTRL piglets. *B. xylanisolvens* was always the least abundant, forming approx. 1% of total microbiota and the remaining *Bacteroides* species were similarly abundant, each forming 5 to 10% of total microbiota (Figure 1B). Intramuscular amoxycillin administration did not affect *Bacteroides* colonisation. The presence of antibiotic resistance genes in the genomes of all used strains was therefore checked and although all strains, except for *B. thetaiotaomicron*, harboured antibiotic resistance genes, only *B*. *fragilis* encoded carbapenemase *cfiA* gene [29] (Table 2). The *tetQ* gene present in *B. vulgatus* differed in sequence from *tetQ* genes, which we recorded in *Bacteroides* sp. using RAST annotation [17] and *B. xylanisolvens* harboured *strA*, *strB* and *sul2* genes common in *E. coli* or *Salmonella* [30,31]. Genes at the same contig upstream and downstream from *strA*, *strB* and *sul2* were manually BLAST-compared with GenBank and these were similar to different *Bacteroides* sequences excluding any sequencing error.

### 3.4. Other Bacteroides Species of Environmental Origin Present in Gut Microbiota of 4-Day-Old Piglets

Absence of a significant difference in the colonisation of experimental and control piglets by *B. vulgatus* indicated that newborn piglets were colonised also by *Bacteroides* of environmental origin (Figure 1D). The additional *Bacteroides* species, which were common in the microbiota of control 4-day-old piglets, included *B. uniformis*, *B. heparinolyticus*, *B. plebeius*, *B. pyogenes*, *Phocaeicola* (*Bacteroides*) *faecicola* and additional sequence variants of *B. fragilis*. The aggregated sum of alternative *Bacteroides* species was lower in gut microbiota of the *Bacteroides* mixture-treated piglets than in the non-treated controls. Having the sequences of each ASV, the distribution of used *Bacteroides* species and those of environmental origin was compared. *B. vulgatus* from the product represented one of the two main branches, while *B. thetaiotaomicron*, *B. xylanisolvens* and *B. fragilis* belonged to another main branch (Figure 1E).

Unlike *Bacteroides*, *Bifido. boum*, *L. ruminis*, *L. salivarius* and *L. mucosae* used in the first experiment did not colonise piglets during the first days of their life (Figure 1B,C).

### 3.5. Consequences of Early Colonisation on the Remaining of Microbiota Members

**Figure 2:**
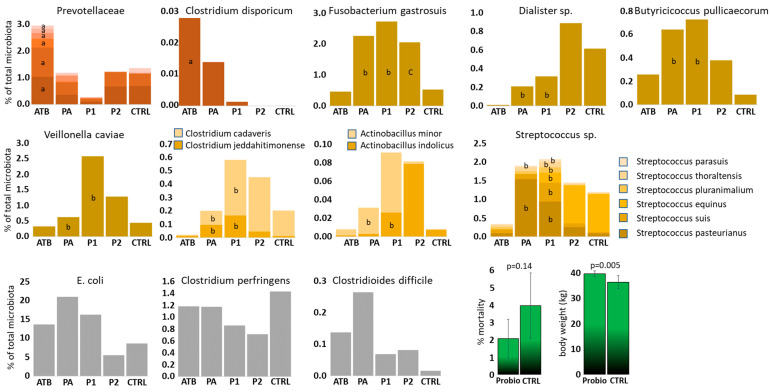
ASVs affected by *Bacteroides* product administration. Brownish, yellow and grey columns, gut microbiota members affected by *Bacteroides* mixture administration. Green columns—consequences of *Bacteroides* mixture treatment of newborn piglets on production parameters during and at the end of pre-fattening period. a—different from P1 group, b—different from ATB group, c—different from CTRL group.

Out of 269 ASVs passing the selection criteria and excluding the strains used for experimental inoculation, 64 ASVs were differently abundant when the microbiota of ATB and P1 piglets was compared, 25 ASVs when the microbiota of ATB and PA piglets was compared and 13 ASVs when the microbiota of CTRL and P2 piglets was compared. This shows a moderate effect of *Bacteroides* mixture administration on the rest of the microbiota and an additional effect of intramuscularly administered amoxycillin. Since some ASVs were differently abundant in multiple comparisons, altogether 73 ASVs were differently abundant in at least one of the comparisons (Table S1). Some ASVs provided conflicting results, e.g., being more abundant in P1 than in ATB piglets and also more abundant in CTRL than in P2 piglets. Such ASVs were excluded from further considerations. Among the remaining ASVs, *Bacteroides* administration promoted colonisation of newborn piglets by *Veillonella caviae*, *Fusobacterium gastrosuis*, *Dialister* sp., *Clostridium jeddahitimonense*, *Clostridium cadaveris*, *Butyricicoccus pullicaecorum*, *Actinobacillus indolicus* and *Actinobacillus minor*. *Bacteroides* mixture resulted also in a higher abundance of multiple *Streptococcus* species incl. *S. pasteurianus*, *S. parasuis*, *S. equinus*, *S. pluranimalium*, *S. thoraltensis* and *S. suis* (Figure 2). On the other hand, administration of *Bacteroides* mixture acted against piglet colonisation by different species from the Prevotellaceae family and *Clostridium disporicum*. In Prevotellaceae and *Clostridium disporicum*, the highest colonisation was recorded in amoxycillin-treated piglets, intermediate in PA piglets and the lowest in P1 piglets indicating the combined effect of antibiotic treatment and *Bacteroides* mixture administration (Figure 2).

Piglet colonisation by *E. coli*, *C. perfringens* and *Cl. difficile*, i.e., the original target species, was not significantly affected by *Bacteroides* mixture administration. Despite this, numerically the highest abundance of *C. perfringens* was recorded in control piglets without any treatment and the lowest in both groups of probiotic treated piglets (Figure 2).

### 3.6. Administration of Bacteroides Mixture and the Effect on Body Weight at the End of the Pre-Fattening Period

In the last experiment, we monitored the performance of the piglets treated with *Bacteroides* mixture till the end of the pre-fattening period 2 months after weaning. There was a lower number of mortalities without reaching statistical significance during the pre-fatting period in *Bacteroides* mixture-treated piglets than in the non-treated controls. Body weight at the end of the pre-fattening period was significantly higher in *Bacteroides* mixture-treated pigs than in non-treated controls (Figure 2).

## 4. Discussion

The gut microbiota consists of hundreds of different bacterial species, which may considerably affect the behaviour of its host [32]. Despite this, bacterial products, which are used to improve the overall status of the host, are limited to a few bacterial genera such as *Lactobacillus*, *Enterococcus*, *Bifidobacterium* or *Bacillus*, while there is no real reason why other non-pathogenic taxa cannot be tested as well. In this study, we therefore tested a mixture of *Bacteroides* species in four independent tests, in one case together with Lactobacilli and *Bifidobacterium*. The tests started with individual litters at a farm from which the used strains originated. The species were selected based on the earlier observation of apparent mutual exclusion of *Bacteroides* sp. and *C. perfringens* [11]. Orally administered *Bacteroides* efficiently colonised piglets from day 1 of life without any adverse effects. On the other hand, when three different *Lactobacillus* species and *Bifido. boum* were included in the first test, these failed to colonise after a single-dose administration. These observations are similar to those in chickens [33,34] and their ability to colonise is likely inversely associated with the ability of prolonged survival of the used bacteria under aerobic conditions [35,36].

Though the administration of *Bacteroides* resulted in efficient colonisation, non-treated control piglets were also positive for *Bacteroides*. This is not surprising as such a colonisation pattern is characteristic for 4-day-old nursed piglets [11]. It can be argued that experimental administration of *Bacteroides* is then of minimal relevance. However, since experimentally administered *Bacteroides* dominated over those of environmental origin, these must have been the first ones to colonise. Treated piglets were therefore colonised earlier and all piglets in the litter were colonised uniformly not leaving any piglet with delayed colonisation by *Bacteroides* species. The presence of additional *Bacteroides* species in the microbiota of control piglets only confirms that experimental administration of *Bacteroides* mimicked and sped up the otherwise natural process of piglet gut colonisation.

*Bacteroides* administration was not effective against original targets such as *C. perfringens*, *Cl. difficile* or *E. coli*. Despite this, we observed that in the case of C. *perfringens*, the piglets with the highest *C. perfringens* abundance belonged to the groups not treated with the *Bacteroides* mixture. The treatment thus prevented *C. perfringens* overgrowth and reduced exposure of littermates to this agent which is shed by highly positive reservoir piglets.

*Bacteroides* treatment affected the intestinal abundance of several other taxa. *Bacteroides* treatment resulted in increased abundance of *Veillonella caviae*. Co-selection of *Veillonella caviae* and *Bacteroides* sp. prevented the formation of attaching and effacing lesions in ligated intestinal loops inoculated with *E. coli* O157:H7 [37] suggesting that these two taxa may indeed protect piglets against pathogens. *Bacteroides* treatment also increased abundance of six different *Streptococcus* species, including pathogenic *S. suis*. This was rather unexpected since the farmers who tested the *Bacteroides* mixture reported reduced need for antibiotic treatment of *S. suis* infections. *Bacteroides* treatment also positively affected the abundance of *Fusobacterium gastrosuis*. *F. gastrosuis* was first identified as pig stomach coloniser [38]. *F. gastrosuis* was also detected as increasing in tonsilar microbiota in *Streptococcus suis*-infected pigs [39]. This suggests that the high abundance of Streptococci due to *Bacteroides*-mixture administration might have caused an increased abundance of *F. gastrosuis*.

Different representatives of Prevotellaceae were more abundant in amoxycillin-treated than in *Bacteroides*-mixture-treated piglets. It is likely that antibiotic therapy, despite intramuscular administration, partially affected gut microbiota development, though not the used *Bacteroides* strains. Piglets with suboptimal gut microbiota were then continuously exposed to Prevotellaceae from their sows, in fact an opposite scenario to the *Bacteroides* administration. Rapid development of gut microbiota towards an adult type in nursed piglets has been shown to have a negative effect on the occurrence of postweaning diarrhoea [14]. Inappropriate microbiota development in the early days of life can then be associated not only with postweaning diarrhoea but also with lower body weight at the end of the pre-fattening period.

Mechanisms why *Bacteroides* may positively affect gut health of newborn piglets have not been addressed in this study. However, *B. thetaiotaomicron* may forage on mucins of host origin, thus facilitating mucin turnover and its availability for other microbiota members [40]. In chickens, bacterial mixtures containing different *Bacteroides* species considerably affected composition of low molecular weight compounds in the caecum which can affect both grow of other microbiota members and host performance [41].

## 5. Conclusions

In this study, we showed that *Bacteroides* sp. can be used as safe probiotics for newborn piglets. Although one must be aware of the occurrence of unusual clones, e.g., as in otherwise commensal *E. coli*, isolates of genus *Bacteroides* can be considered as safe for that sensitive model as newborn piglets are.

## Figures and Tables

**Table 1:** List of strains used in individual experiments of this study.

	Farm A	Farm A	Farm A	Farm B
	Exp 1	Exp 2	Exp 3	Exp 4
*Bacteroides thetaiotaomicron* An878	✓			
*Bacteroides thetaiotaomicron* ET42		✓	✓	✓
*Bacteroides vulgatus* An905	✓	✓	✓	✓
*Bacteroides xylanisolvens* An931	✓	✓	✓	✓
*Bacteroides fragilis* ET48		✓	✓	✓
*Bifidobacterium boum* An918	✓			
*Lactobacillus ruminis* An917	✓			
*Lactobacillus salivarius* An879	✓			
*Lactobacillus mucosae* An939	✓			

**Table 2:** Presence of antibiotic resistance genes in the genomes of strains used in this study.

	n.ATB Genes *	tetQ ^#^	cfiA	mefA	strA	strB	sul2	aph3	aph6	ant6	tetM	ermB	tetW
*Bacteroides thetaiotaomicron* ET42	0												
*Bacteroides thetaiotaomicron* An878	1	✓											
*Bacteroides xylanisolvens* An931	6			✓	✓	✓	✓	✓	✓				
*Bacteroides vulgatus* An905	1	✓											
*Bacteroides fragilis* ET48	1		✓										
*Lactobacillus salivarius* An879	2									✓	✓		
*Lactobacillus ruminis* An917	3									✓	✓	✓	
*Lactobacillus mucosae* An939	1												✓
*Bifidobacterium boum* An918	1												✓

* number of different antibiotic resistance genes. ^#^ tetQ and the remaining columns, individual antibiotic resistance genes detected in the genomes of used strains.

## Data Availability

Raw sequencing data have been deposited in GenBank under accession number PRJNA1309669.

## Supplementary Materials

The following supporting information can be downloaded at: https://www.mdpi.com/article/10.3390/microorganisms13102356/s1, Table S1: List of ASVs recorded in this study.
